# Gestational and neonatal outcomes of women with partial Dunnigan lipodystrophy

**DOI:** 10.3389/fendo.2024.1359025

**Published:** 2024-04-03

**Authors:** Cynthia M. Valerio, Raquel Beatriz Gonçalves Muniz, Luiz F. Viola, Gabriela Bartzen Pereira, Rodrigo Oliveira Moreira, Marise Ribeiro de Sousa Berriel, Renan Magalhães Montenegro Júnior, Amélio F. Godoy-Matos, Lenita Zajdenverg

**Affiliations:** ^1^ Department of Metabolism, Institute of Diabetes and Endocrinology of Rio de Janeiro (IEDE), Rio de Janeiro, Brazil; ^2^ Brazilian Group for the Study of Inherited and Acquired Lipodystrophies (BRAZLIPO), Fortaleza, Brazil; ^3^ Diabetes and Endocrinology Center (CEDERO), Rondonópolis, Brazil; ^4^ Clinical Research Unit, Walter Cantídio University Hospital, Federal University of Ceará/EBSERH, Fortaleza, Brazil; ^5^ Internal Medicine Department - Nutrology and Diabetes Session, Federal University of Rio de Janeiro (UFRJ), Rio de Janeiro, Brazil

**Keywords:** lipodystrophy, pregnancy, FPLD2, Dunnigan, gestational diabetes mellitus

## Abstract

**Introduction:**

Lipodystrophies are a group of disorders characterized by selective and variable loss of adipose tissue, which can result in an increased risk of insulin resistance and its associated complications. Women with lipodystrophy often have a high frequency of polycystic ovary syndrome (PCOS) and may experience gynecological and obstetric complications. The objective of this study was to describe the gestational outcomes of patients with familial partial lipodystrophy type 2 (FPLD2) at a reference center with the aim of improving the understanding and management of pregnant women affected by this condition.

**Methods:**

This was a retrospective analysis of data obtained from questionnaires regarding past pregnancies and a review of medical records from the beginning of follow-up in outpatient clinics.

**Results:**

All women diagnosed with FPLD2 who had previously become pregnant were included in this study (n=8). The women in the study experienced pregnancies between the ages of 14 and 38 years, with an average of 1.75 children per woman. The pregnancies in question were either the result of successful conception within 12 months of attempting to conceive or unplanned pregnancies. During pregnancy, two women (25%) were diagnosed with gestational diabetes mellitus (GDM), one (12.5%) with gestational hypothyroidism, and one (12.5%) with preeclampsia. Among the 17 pregnancies, two miscarriages (11.8%) occurred, and five cases (29.4%) of macrosomia were observed. Four instances of premature birth and an equal number of neonatal hypoglycemia cases were recorded. The reported neonatal complications included an unspecified malformation, respiratory infection, and two neonatal deaths related to heart malformation and respiratory distress syndrome.

**Conclusion:**

Our data showed a high frequency of fetal complications in women with FPLD2. However, no instances of infertility or prolonged attempts to conceive have been reported, highlighting the significance of employing effective contraception strategies to plan pregnancies at optimal times for managing metabolic comorbidities.

## 1 Introduction

Lipodystrophies are uncommon conditions characterized by a selective and variable loss of adipose tissue, which can lead to an increased risk of insulin resistance and its associated complications. These disorders can manifest in various forms, which are broadly categorized as familial or acquired, contingent upon their etiology; and as generalized or partial, depending on the extent of adipose tissue loss. As a result, the clinical presentation and age at which lipodystrophies occur can vary widely, leading to different challenges in diagnosis and management (1, 2).

Partial lipodystrophies have a higher prevalence than previously thought, estimated in up to one case for every 7000 people (3). The most common type is the Dunnigan variant or familial partial lipodystrophy type 2 (FPLD2), which is inherited in an autosomal dominant manner and is caused by variants of the *LMNA* gene on chromosome 1q21-22. Clinical manifestations typically appear after puberty and include reduced fat distribution in the limbs and trunk along with excess fat accumulation around the neck and face. Other clinical features may include prominent musculature, enlarged veins (phlebomegaly), and acanthosis nigricans (4–6).

Changes in the secretory and metabolic processes of adipocytes due to the lack of functional subcutaneous adipose tissue in lipodystrophy syndromes, result in multiple metabolic alterations. These include insulin resistance, metabolic dysfunction-associated steatotic liver disease (MASLD), myocardial issues, endothelial dysfunction, and pancreatic beta cell abnormalities (7). Phenotypic characteristics associated with *LMNA* gene variants are typically observed in females during puberty, when there is a high presence of sex hormones, although some changes may also occur before puberty begins (8, 9).

Women with lipodystrophy have a higher frequency of polycystic ovary syndrome (PCOS) and may have gynecological and obstetric complications such as spontaneous miscarriages, infertility, gestational diabetes mellitus (GDM), hypertensive disorders of pregnancy, newborns with macrosomia, and fetal death compared to both the general population and those with PCOS (1). Additionally, these patients, especially those with FPLD2, are described as having fewer living children than their unaffected relatives (1, 5). Given the limited information in the medical literature regarding gestational and neonatal outcomes of FPLD2, this study aimed to describe the gestational outcomes of patients with FPLD2 at a lipodystrophy reference center, with the aim of improving the understanding and management of pregnant women affected by this condition.

## 2 Methods

This study involved a retrospective analysis of data obtained from questionnaires and a review of medical records from the beginning of data collection in January 2022 until February 2024. This study focuses on FPLD2 patients with prior pregnancies who are undergoing outpatient follow-up at the State Institute of Diabetes and Endocrinology, Luiz Capriglione, a public hospital in Rio de Janeiro, Brazil. Ethical approval was granted by the hospital’s ethics committee and all participants provided informed consent for their involvement (51619421.5.0000.5266). This research encompassed a comprehensive review of the participants’ medical records to gather details about their demographic characteristics, medical history, pregnancies, and neonatal outcomes.

The collected data included details on the current age of the participants and comorbidities, including the assessment of the highest triglyceride level measured enzymatically, glycated hemoglobin levels using an HPLC assay, and complications associated with diabetes mellitus. As the medical records of the pregnancies were not available, a questionnaire specifically addressing pregnancy-related information was administered (see Appendix 1). The questionnaire comprised questions on the number of pregnancies, age at pregnancy, time to conception, comorbidities diagnosed before pregnancy, gestational age at delivery, presence of GDM or other complications during pregnancy, birth weight of the newborn, and neonatal complications.

## 3 Results

Among 14 patients diagnosed with FPLD2 undergoing medical follow-up in our service, this study included 8 who had previously become pregnant, resulting in a total of 17 pregnancies, with one twin gestation. All individuals were carriers of pathogenic *LMNA* variants affecting exons 8 and 11. The variants detected were p.R482W, p.R482Q, p.N466D, and p.R582C, and one patient exhibited both the p.R482W variant and another in the lipoprotein lipase (*LPL*) gene. The clinical characteristics of the study participants are summarized in **Table 1**.

**Table 1:** Demographic, genetic, and clinical characteristics of the study participants.

PATIENTS	P1	P2	P3	P4	P5	P6	P7	P8
Current Age (years)	39	41	62	60	52	44	42	54
Variant	*LMNA* p.N466D	*LMNA* p.R582C	*LMNA* p.R482Q	*LMNA* p.R482Q	*LMNA* p.R482W *LPL* p.N318S	*LMNA* p.R482W	*LMNA* p.R482W	*LMNA* p.R482W
Diabetes Mellitus (current)	Yes	Yes	Yes	Yes	Yes	Yes	Yes	No
Age at diabetes onset (years)	18	27	46	32	37	29	26	-
Number of pregnancies or parity	1	3	3	1	1	5	2 (including a twin gestation)	1
Age at pregnancies	24	16 to 36	35 to 38	31	29	14 to 33	25 and 35	33
Current diagnosis of MASLD	Yes	Yes	Yes	Yes	Yes	Yes	Yes	Yes
History of Pancreatitis	Yes	No	No	No	No	No	No	No
Highest triglyceride (mg/dL)	6397	3505	324	423	2127	574	501	276
Age of highest triglyceride (in years)	26	37	49	41	50	31	40	53
Chronic hypertension	Yes	Yes	Yes	Yes	Yes	Yes	Yes	Yes
History of PCOS	Yes	No	Yes	No	No	Yes	Yes	No

MASLD, metabolic dysfunction-associated steatotic liver disease; PCOS, polycystic ovary syndrome.

Pathogenic variants detected in the patients.

Coloured text denotes pathogenic variants detected in the patients.

The age range for pregnancies in our study population was broad, varying from 14 to 38 years. On an average, each woman had 1.75 living children. The pregnancies were either the result of successful conception within 12 months of attempting to conceive, or unplanned pregnancies. We documented 17 pregnancies, of which 15 resulted in live births, yielding 16 neonates due to one set of twins. The remaining two pregnancies (11.8%) ended in miscarriages, affecting two patients who had not been previously diagnosed with T2DM at the ages of 18 and 30, respectively. Among live births, there were two neonatal deaths, each attributed to different causes. One death was due to congenital heart defects that occurred in a neonate whose mother had a history of T2DM. The other involved respiratory distress syndrome in a child born to a mother diagnosed with GDM during pregnancy, which then progressed to T2DM in the following year.

Half of the female patients in our study, specifically four out of eight, had preexisting medical conditions prior to at least one pregnancy. These conditions include T2DM, Systemic Arterial Hypertension (SAH), and PCOS. During pregnancy, two women developed gestational diabetes mellitus (25%) and one experienced preeclampsia. Notably, 31.25% (5 out of 16) of live newborns were large for gestational age, with birth weights above the 90th percentile. Furthermore, 26.7% (4 out of 15) of childbirth events were preterm deliveries, including twin gestation.

Four of the 16 neonates (25%) experienced neonatal complications, including neonatal hypoglycemia in four newborns (25%), respiratory distress syndrome in two (12.5%), and congenital heart malformations and unspecified malformations, each occurring in one instance. Maternal and neonatal outcomes are described in **Table 2**.

**Table 2:** Maternal and neonatal outcomes in patients with FPLD2.

PATIENTS	P1	P2			P3			P4	P5	P6					P7		P8
Time to conceive in months or unplanned pregnancy (UP)	UP	UP			4			12	UP	UP					UP		UP
Pregnancy number	1^st^	1st	2nd	3rd	1st	2nd	3rd	1st	1st	1st	2nd	3rd	4th	5th	1st	2nd (twin)	1st
Age at pregnancy (in years)	24	16	18	36	30	35	38	31	29	14	16	27	34	35	25	35	33
Miscarriage	No	No	Yes	No	Yes	No	No	No	No	No	No	No	No	No	No	No	No
GDM	No	No	–	No	–	No	No	No	No	No	Yes	Yes	No	No	Yes	No	No
Preeclampsia	No	Yes	–	Yes	–	No	No	No	No	No	No	No	No	No	No	No	No
Gestational hypothyroidism	Yes	No	–	No	–	No	No	No	No	No	No	No	No	No	No	No	No
Macrosomia	Yes	No	–	No	–	No	Yes	No	Yes	No	No	Yes	Yes	No	No	No	No
Neonatal hypoglycemia	No	No	–	Yes	–	No	No	No	No	No	No	Yes	Yes	Yes	No	No	No
Prematurity	No	Yes	–	Yes	–	No	No	No	No	No	No	No	No	No	Yes	Yes	No
Malformation	No	No	–	Yes	–	No	No	No	No	No	No	No	No	Yes	No	No	No
Neonatal death	No	No	–	No	–	No	No	No	No	No	No	No	No	Yes	Yes	No	No

GDM, Gestational Diabetes Mellitus; UP, unplanned pregnancy.

The values are: 4 and 12 months.

In our study, one individual (referred to as patient 2), who had three pregnancies, experienced two instances of preeclampsia. These complications led to preterm delivery. During her second attempt at pregnancy, she experienced miscarriage. In her subsequent pregnancy at the age of 36 years, she encountered several challenges. In addition to preeclampsia, she was previously diagnosed with T2DM at 27 years of age. This pregnancy also resulted in preterm delivery, which involved complications such as unspecified neonatal malformations and hypoglycemia in the newborn.

Of the five pregnancies that we documented with macrosomia, two were in mothers who had a prior diagnosis of T2DM, and one involved GDM. Notably, the remaining two cases involved mothers who had no prior diagnosis of either T2DM or GDM at the beginning of their pregnancies. However, both of these women, aged 29 and 38 years at the time of their pregnancies, were later diagnosed with T2DM.

## 4 Discussion

Our research identified a higher occurrence of adverse maternal-fetal outcomes, such as spontaneous abortions, GDM, preeclampsia, and macrosomia, in women diagnosed with FPLD2 than in those without this condition (1, 10–16). These complications encompass GDM, preeclampsia, fetal macrosomia, neonatal hypoglycemia, among other fetal complications. Conditions such as diabetes mellitus, chronic hypertension, and PCOS, which are prevalent in patients with FPLD2, may have contributed to these unfavorable outcomes. These results indicate that women with FPLD2 have heightened susceptibility to complications during pregnancy, which can impact maternal, fetal, and neonatal health outcomes.

In comparison to studies conducted in the general Brazilian population using primary data and laboratory measurements with the International Association of Diabetes and Pregnancy Study Groups criteria, we observed a higher prevalence of GDM in those with FPLD2 (25% *vs*. 18.0%) (12). Non-pregnant Brazilian women have been reported to have a diabetes mellitus prevalence of 8.4% (13), which is significantly lower than the 87.5% prevalence we documented.

Regarding pregnancy outcomes, one study found that the combined prevalence of preeclampsia in the general Brazilian population was 6.7% (14). The rate of macrosomic births was 5.3% between 2001 and 2010 and 5.1% between 2012 and 2014 (15). Conversely, in our study group, 31.25% of newborns were macrosomic, and preeclampsia affected 11.8% of the cases, nearly double that observed in the Brazilian population. Additionally, the rate of miscarriages in our cohort was 11.8%, which closely aligns with the general estimate of 14% for the Brazilian population (16).

Previous studies have shown that women with lipodystrophy, particularly those with Dunnigan-type familial partial lipodystrophy, are more susceptible to metabolic complications than men are, indicating the potential influence of estrogen on disease expression (17). Garg suggested that the underlying genetic defect may involve a lack of a protein or receptor in adipose tissue that is responsive to steroids, which is crucial for stimulating growth and preserving subcutaneous adipose tissue in key areas such as the extremities, hips, and trunk during puberty (17). Hormonal and metabolic disruptions linked to lipodystrophy can negatively affect pregnancy outcomes. Further investigation is needed to understand how lipodystrophy affects pregnancy results and to create personalized strategies for managing pregnant women with lipodystrophy.

During pregnancy, individuals with lipodystrophy may experience worsening insulin resistance, making it challenging to manage diabetes and posing a risk to the fetus (1). In the second half of pregnancy, various factors, such as impaired insulin response in peripheral tissues, decreased hepatic suppression of glucose production, decreased insulin-stimulated uptake in skeletal muscle, increased secretion of hormones, cytokines, and adipokines, or the release of other substances from the placenta to the maternal circulation, contribute to insulin resistance. Consequently, patients with beta cell impairment may develop GDM, whereas those with preexisting DM often require an increase in insulin supply during this stage (18).

In our series, among the five pregnancies related to macrosomia, two were associated with previous T2DM, one with GDM, and two were in patients later diagnosed with T2DM. Additionally, patients diagnosed with DM, GDM, and PCOS, which are commonly recognized as predictors of obstetric and fetal complications, experienced adverse maternal and fetal outcomes, including spontaneous abortion, malformation, and neonatal hypoglycemia.

A considerable proportion of our sample was affected by hypertensive disorders during their lifetimes. It is well established that both DM and hypertension are prominent risk factors for preeclampsia, with a higher relative risk observed in patients with poorly controlled DM. Additionally, studies indicate that approximately 20% of individuals with a history of hypertensive disorders during pregnancy subsequently develop superimposed preeclampsia (19).

The current age of the individuals ranged from 39 to 60 years. Of these patients, only one aged 54 years had not been diagnosed with diabetes mellitus. Within a timeframe of one–two years following pregnancy, three patients were subsequently diagnosed with T2DM, of whom two had a pregnancy with GDM. This suggests a natural progression in most cases of lipodystrophy. In FPLD2, hypertriglyceridemia typically occurs early in life after puberty and DM generally develops later in adulthood, although some cases manifest early after puberty (9).

DM in lipodystrophic patients is characterized by severe insulin resistance and high levels of circulating insulin, leading to elevated triglyceride levels. Managing hyperglycemia in these patients is challenging and often requires a combination of oral antidiabetic agents and large doses of insulin, resulting in early onset complications associated with diabetes (5, 17). Age is considered a risk factor for GDM even in the general population. Given the progressive nature of metabolic changes in lipodystrophy, it can be inferred that pregnancies occurring at an older age will likely have increased maternal and fetal risks (20). In our study, most pregnancies in women aged > 30 years had adverse outcomes.

Our findings align with the existing literature on pregnancy and lipodystrophy, indicating increased insulin resistance, metabolic changes, and a higher prevalence of fetal complications. In a study by Vantyghem et al., which included 14 women with FPLD2, the frequency of PCOS was higher than 50% and obstetric complications were higher than those in the general population. This includes a GDM frequency of 30%, spontaneous abortions of 50%, and preeclampsia and fetal death exceeding 10% (1).

Another study examined seven individuals with partial lipodystrophy, two of whom had *LMNA* and *PPARG* variants, four with unknown genetic causes, and one patient with generalized lipodystrophy (21). A total of 21 pregnancies were identified, with 67% occurring spontaneously and 33% requiring fertility treatment. Six patients had ten pregnancies that resulted in live births. Among these 10 live births, 7 were term deliveries and 3 were preterm deliveries. All ten pregnancies resulting in live births were complicated by diabetes.

In contrast, 25% of our patients had a history of GDM. Among the 17 pregnancies, 11.8% ended in spontaneous abortion, whereas 29.4% resulted in neonates with macrosomia. Additionally, over 20% of pregnancies resulted in preterm delivery, along with other obstetric and neonatal complications. However, unlike the studies by Vantyghem and Neal (1, 21), which reported fertility problems at a rate of approximately 30%, we did not observe any cases of infertility or extended efforts to conceive in women with a history of pregnancy and FPLD2, including those previously diagnosed with PCOS. This highlights the significance of contraception in planning pregnancies at an optimal time for managing metabolic comorbidities.

It should be noted that when advising on contraception, it is important to carefully consider the use of oral contraceptives containing ethinyl estradiol and/or progestagens, given their potential to worsen pre-existing cardiovascular conditions and the risk of pancreatitis (22).

Our study has some limitations. Since the study was conducted retrospectively, there were challenges in gathering certain data. Additionally, the patients received obstetric care from another service. However, these limitations do not undermine the validity of our results, as this pathology is rare, and there is limited literature available on it.

Maternal-fetal monitoring of patients with FPL must be intensive and categorized as high-risk prenatal care, given the high frequency of perinatal complications described in patients with FPLD2. Therefore, it is crucial for healthcare providers to closely monitor and manage these patients during pregnancy to minimize the risk of adverse outcomes for both the mother and the fetus.

This study provides valuable insights into gestational and neonatal outcomes in patients with FPLD2 and highlights the importance of further research in this area to better understand the implications of lipodystrophy on pregnancy and develop tailored management strategies.

## Data availability statement

The original contributions presented in the study are included in the article/supplementary material, further inquiries can be directed to the corresponding author/s.

## Ethics statement

The studies involving humans were approved by Institute of Diabetes and Endocrinology of Rio de Janeiro (IEDE). The studies were conducted in accordance with the local legislation and institutional requirements. The participants provided their written informed consent to participate in this study. Written informed consent was obtained from the individual(s) for the publication of any potentially identifiable images or data included in this article.

## Author contributions

CV: Writing – review & editing, Writing – original draft, Methodology, Investigation, Data curation, Conceptualization. RGM: Writing – review & editing, Writing – original draft, Methodology, Data curation, Conceptualization. LV: Writing – review & editing, Writing – original draft, Software, Methodology, Data curation. GP: Writing – review & editing, Writing – original draft, Methodology, Data curation. RdOM: Writing – review & editing, Writing – original draft, Project administration, Conceptualization. MB: Writing – review & editing, Writing – original draft, Data curation. RMM: Writing – review & editing, Writing – original draft, Supervision, Methodology. AM: Writing – review & editing, Writing – original draft, Supervision, Project administration. LZ: Writing – review & editing, Writing – original draft, Supervision, Project administration.

## Appendix 1

Questionnaire

1. How many pregnancies have you had? How long did it take to conceive (months)?

2. Have you experienced any miscarriages? If yes, how many and at what gestational age?

3. Did you have any preexisting conditions before trying to conceive? These include diabetes mellitus, systemic arterial hypertension, and polycystic ovary syndrome (PCOS).

4. What complications did you experience during your pregnancy? Examples include gestational diabetes mellitus, eclampsia, and preeclampsia.

5. If you had diabetes or gestational diabetes mellitus, were insulin injections necessary to control your condition? What is the total dose per kilogram of body weight?

6. Describe the pattern of your menstrual cycles before pregnancy: were they regular?

Regarding the newborn:

7. How old was your newborn in weeks and days?

8. What was the birth weight of your baby?

9. Were there any fetal complications? Was admission to an intensive care unit necessary, and if so, for what reason?

10. Has the newborn experienced neonatal hypoglycemia?

## References

[1] Vantyghem MC Vincent-Desplanques D DeFrance-Faivre F Capeau J Fermon C Valat AS. Fertility and obstetrical complications in women with *LMNA* -related familial partial lipodystrophy. J Clin Endocrinol Metab. (2008) 93:2223–9. doi: 10.1210/jc.2007-2521 18364375

[2] Vantyghem MC Balavoine AS Douillard C DeFrance F Dieudonne L Mouton F. How to diagnose a lipodystrophy syndrome. Ann Endocrinol. (2012) 73:170–89. doi: 10.1016/j.ando.2012.04.010 22748602

[3] Fourman LT Grinspoon SK. Approach to the patient with lipodystrophy. J Clin Endocrinol Metab. (2022) 107:1714–26. doi: 10.1210/clinem/dgac079 PMC9113814 35137140

[4] Hussain I Garg A. Lipodystrophy syndromes. Endocrinol Metab Clin North Am. (2016) 45:783–97. doi: 10.1016/j.ecl.2016.06.012 PMC7590232 27823605

[5] Brown RJ Araujo-Vilar D Cheung PT Dunger D Garg A Jack M. The diagnosis and management of lipodystrophy syndromes: A multi-society practice guideline. J Clin Endocrinol Metab. (2016) 101:4500–11. doi: 10.1210/jc.2016-2466 PMC5155679 27710244

[6] Zhong ZX Harris J Wilber E Gorman S Savage DB O’Rahilly S. Describing the natural history of clinical, biochemical and radiological outcomes of children with familial partial lipodystrophy type 2 (FPLD2) from the United Kingdom: A retrospective case series. Clin Endocrinol (Oxf). (2022) 97:755–62. doi: 10.1111/cen.14806 PMC9804585 35920656

[7] Zammouri J Vatier C Capel E Auclair M Storey-London C Bismuth E. Molecular and cellular bases of lipodystrophy syndromes. Front Endocrinol. (2022) 12:803189. doi: 10.3389/fendo.2021.803189 PMC8763341 35046902

[8] Bagias C Xiarchou A Bargiota A Tigas S. Familial partial lipodystrophy (FPLD): recent insights. Diabetes Metab Syndr Obes Targets Ther. (2020) 13:1531–44. doi: 10.2147/DMSO.S206053 PMC7224169 32440182

[9] Mosbah H Donadille B Vatier C Janmaat S Atlan M Badens C. Dunnigan lipodystrophy syndrome: French National Diagnosis and Care Protocol (PNDS; Protocole National de Diagnostic et de Soins). Orphanet J Rare Dis. (2022) 17:170. doi: 10.1186/s13023-022-02308-7 35440056 PMC9019936

[10] Besci O Foss De Freitas MC Guidorizzi NR Guler MC Gilio D Maung JN. Deciphering the clinical presentations in *LMNA*-related lipodystrophy: report of 115 cases and a systematic review. J Clin Endocrinol Metab. (2024) 109(3):e1204–24. doi: 10.1210/clinem/dgad606 PMC10876415 37843397

[11] Cabré A Lázaro I Cofán M Jarauta E Plana N Garcia-Otín AL. FABP4 plasma levels are increased in familial combined hyperlipidemia. J Lipid Res. (2010) 51:1173–8. doi: 10.1194/jlr.M900066 PMC2853443 20388924

[12] Iser BPM Stein C Alves LF Carvalho MLS Espinoza SAR Schmidt MI. A portrait of gestational diabetes mellitus in Brazil: A systematic review and meta-analysis. Arch Endocrinol Metab. (2023) 67:e220521. doi: 10.20945/2359-4292-2022-0521 37856706

[13] Guida JPS Andrade BG Pissinatti LGF Rodrigues BF Hartman CA Costa ML. Prevalence of Preeclampsia in Brazil: An Integrative Review. Rev Bras Ginecol Obstet. (2022) 44:(7)686–91. doi: 10.1055/s-0042-1742680 PMC9948112 35139578

[14] Reis RCPD Duncan BB Malta DC Iser BPM Schmidt MI. Evolution of diabetes in Brazil: prevalence data from the 2013 and 2019 Brazilian National Health Survey [published correction appears in Cad Saude Publica. Cad Saude Publica. (2022) 38Suppl 1:e00149321. doi: 10.1590/0102-311X00149321 35544921

[15] Nascimento MID Pereira DF Lopata C Oliveira CLF Moura AA Mattos MJS. Trends in the Prevalence of Live Macrosomic Newborns According to Gestational Age Strata, in Brazil, 2001-2010, and 2012-2014. Tendências na prevalência de recém-nascidos vivos macrossômicos, estratificadas por idade gestacional, Brasil, 2001–2010 e 2012–2014. Rev Bras Ginecol Obstet. (2017) 39:376–83. doi: 10.1055/s-0037-1604266 PMC10316946 28783857

[16] Cecatti JG Guerra GV Sousa MH Menezes GM. Aborto no Brasil: um enfoque demográfico [Abortion in Brazil: a demographic approach]. Rev Bras Ginecol Obstet. (2010) 32:105–11. doi: 10.1590/s0100-72032010000300002 20512256

[17] Garg A. Lipodystrophies. Am J Med. (2000) 108:143–52. doi: 10.1016/s0002-9343(99)00414-3 11126308

[18] Kampmann U Knorr S Fuglsang J Ovesen P. Determinants of maternal insulin resistance during pregnancy: an updated overview. J Diabetes Res. (2019) 2019:1–9. doi: 10.1155/2019/5320156 PMC6885766 31828161

[19] Magee LA Pels A Helewa M Rey E Von Dadelszen P. Diagnosis, evaluation, and management of the hypertensive disorders of pregnancy. Pregnancy Hypertens Int J Womens Cardiovasc Health. (2014) 4:105–45. doi: 10.1016/j.preghy.2014.01.003 26104418

[20] Sweeting A Wong J Murphy HR Ross GP. A clinical update on gestational diabetes mellitus. Endocr Rev. (2022) 43:763–93. doi: 10.1210/endrev/bnac003 PMC9512153 35041752

[21] Neal TJ Startzell MS Cochran E Lightbourne M Brown RJ. THU301 natural history of pregnancy and pregnancy outcomes in subjects with lipodystrophy. J Endocr Soc. (2023) 7:bvad114.736. doi: 10.1210/jendso/bvad114.736

[22] Sollier C Vatier C Capel E Lascols O Auclair M Janmaat S. Lipodystrophic syndromes: From diagnosis to treatment. Ann Endocrinol. (2020) 81:51–60. doi: 10.1016/j.ando.2019.10.003 31982105

